# The stoneflies (Plecoptera) of West Virginia: a new occurrence dataset

**DOI:** 10.3897/BDJ.14.e185229

**Published:** 2026-06-16

**Authors:** Theodore Z. Cook, R. Edward DeWalt, Scott A. Grubbs, Phillip N. Hogan, Lily Veronica Hart, Shawn M. Clark, Richard W. Baumann, David K. Burton, Luke W. Myers

**Affiliations:** 1 Department of Entomology, University of Illinois, Urbana-Champaign, IL, United States of America Department of Entomology, University of Illinois Urbana-Champaign, IL United States of America; 2 Illinois Natural History Survey, University of Illinois, Urbana-Champaign, IL, United States of America Illinois Natural History Survey, University of Illinois Urbana-Champaign, IL United States of America; 3 Western Kentucky University, Bowling Green, KY, United States of America Western Kentucky University Bowling Green, KY United States of America; 4 Brigham Young University (retired), Provo, UT, United States of America Brigham Young University (retired) Provo, UT United States of America; 5 Canadian National Collection, Ottawa, Canada Canadian National Collection Ottawa Canada; 6 SUNY, Plattsburgh, NY, United States of America SUNY Plattsburgh, NY United States of America

**Keywords:** stoneflies, West Virginia, new state records, taxonomy, collections

## Abstract

**Background:**

Stoneflies are a relatively small order of insects comprising over 4,200 extant, described species. They have long been recognised as valuable indicators of water quality, since nymphs are dependent on clean water conditions and are sensitive to the effects of sedimentation and pollution. It is also well-documented that stonefly species around the world have suffered contractions in range, leading, in some cases, to local extirpation or extinction. West Virginia is home to high stonefly species richness relative to most other USA (United States of America) states. Despite this diversity, the available literature is scattered and occurrence datasets supported by voucher specimens are non-existent. Funding from the U.S. Fish and Wildlife Service and a consortium of northeastern USA states has provided an opportunity to document the stonefly fauna of West Virginia, especially for state and regional species of conservation interest, by gathering specimen-based occurrence data from museums, collecting new specimens statewide, and compiling trusted literature records into a single statewide dataset. Our hope is that the records presented in this data paper will further the understanding of stonefly species distribution in West Virginia and the eastern USA in general, and provide conservation agencies with usable temporal and locality data. These data will be used as the basis of an upcoming distribution atlas and annotated checklist of the stoneflies of West Virginia.

**New information:**

Our dataset brings together over 4,500 stonefly records from all 55 counties in West Virginia. These records, spanning the previous 134 years, have been contributed by 17 institutions and personal collections. Through new collecting and the examination of museum specimens, we have been able to verify the presence of 150 species, with representatives from all nine eastern North American families. Fourteen species previously recorded from West Virginia, comprising seven Perlodidae, six Perlidae and one Chloroperlidae, have not been recovered. Most of these absences can be accounted for by changes in taxonomic nomenclature. In addition to 140 species already known from the state, we report the presence of 11 new state records, including four Perlidae, three Perlodidae, two Capniidae, one Leuctridae and one Nemouridae.

## Introduction

Stoneflies (Plecoptera) are a hemimetabolous taxon of aquatic insects that are widely spread across the globe (DeWalt and Ower 2019). Five families are limited exclusively to the Southern Hemisphere, while the other 12 families are at their most diverse in the Holarctic Region (Letsch et al. 2021, South et al. 2021a, South et al. 2021b, Eichert et al. 2025). Plecoptera nymphs can be found in many freshwater ecosystems including lakes and aquifers, as well as some terrestrial habitats such as caves (López-Rodríguez and Tierno de Figueroa 2012, South et al. 2021b). However, most species inhabit cool or coldwater lotic systems (DeWalt and Ower 2019). Many different functional feeding groups are represented by Plecoptera taxa, including shredders, scrapers and predators (Merritt et al. 2017, Tierno de Figueroa and López-Rodríguez 2019). Most species are broadly intolerant of chemical pollution, siltation and heightened water temperature and, as a result, stoneflies tend to be one of the first macroinvertebrate groups to disappear from a degraded stream system (DeWalt and Ower 2019). Such sensitivities to disturbance have resulted in significant species losses in the Midwestern states of Illinois and Ohio (DeWalt et al. 2005, DeWalt et al. 2012).

The state of West Virginia, United States of America (USA), contains an especially diverse assemblage of stoneflies. Major river drainages, such as the Ohio, Kanawha and Potomac watersheds, in addition to the bisecting Allegheny Mountains, give the state a high degree of habitat heterogeneity. Thus, West Virginia has a long history of stonefly collection within its borders. Noteworthy past collectors have included T. H. Frison, O. S. Flint, H. B. N. Hynes, R. W. Baumann, K. W. Stewart, S. M. Clark, M. B. Griffith, D. R. Smith, D. C. Tarter, B. C. Kondratieff and R. F. Kirchner. Despite this intensive collecting, no statewide dataset has yet been published. Tarter and Kirchner (1980) published an initial state checklist tallying 106 species. Tarter and Nelson improved upon this figure in two subsequent checklists, which together recorded a total of 140 species (Tarter and Nelson 2006, Tarter and Nelson 2010). None of these publications included detailed event data, instead listing counties in which the specimens were found and the relevant repositories (e.g. Marshall University, West Virginia University, US Army Corps of Engineers and others). Many of these specimens have since been lost. Thus, their presence and specific locality often cannot be confirmed without further collecting.

Later publications describing new species raised the number of recorded species to 150 (Kondratieff and Kirchner 2009, Grubbs and DeWalt 2012, Szczytko and Kondratieff 2015, Verdone and Kondratieff 2016, Grubbs and Baumann 2019, Verdone et al. 2025). However, no single resource has heretofore existed that brings together detailed specimen data of all known West Virginian species.

### Dataset Description

The publication of this paper follows that of six other statewide treatments of stonefly fauna (DeWalt et al. 2012, DeWalt et al. 2016, Newman et al. 2021, Hogan and Grubbs 2022, Hart et al. 2025, Myers et al. 2025). It is indebted to them for its similar methodology.

In this paper, we present a newly-compiled dataset of 4,612 stonefly records from West Virginia (Cook et al. 2026). We document the presence of 150 species, including 11 new state records. Valid name usage was confirmed by comparison to names found in Plecoptera Species File (DeWalt et al. 2025). Following new collecting and the re-examination of preserved specimens, we have substantiated the presence of three species previously removed from the state list (Tarter and Nelson 2006): *Alloperla idei* (Ricker, 1935), *Perlesta placida* (Hagen, 1861) and *Yugus bulbosus* (Frison, 1942). Fourteen species from Tarter and Nelson (2006) and Tarter and Nelson 2010 have not been recovered: *Acroneuria perplexa* Frison 1937, *Perlesta cinctipes* (Banks, 1905), *Perlesta frisoni* Banks, 1948, *Perlesta lagoi* Stark, 1989, *Perlesta puttmanni* Kondratieff & Kirchner, 2003, *Perlesta shubuta* Stark, 1989, *Sweltsa onkos* (Ricker, 1936) and the seven *Isoperla* species *Isoperla cotta* Ricker, 1952, *Isoperla gibbsae* Harper, 1971, *Isoperla marlynia* Needham & Claassen, 1925, *Isoperla namata* Frison, 1942, *Isoperla similis* (Hagen, 1961), *Isoperla slossonae* (Banks, 1911) and *Isoperla transmarina* (Newman, 1838). A fifteenth species, *Acroneuria kirchneri* (Stark & Kondratieff, 2004), was synonymised with *Acroneuria kosztarabi* (Kondratieff & Kirchner, 1993) and is no longer considered a valid species (Verdone et al. 2022).

*Perlesta cinctipes* is no longer thought to occur east of Illinois, and it is likely that specimens recorded by Tarter and Nelson (2006) from Monongalia County represented *P. armitagei* Grubbs and DeWalt, 2018 or other *Perlesta* (DeWalt and Grubbs 2011, Grubbs and DeWalt 2018). Similarly, *Perlesta shubuta* is now believed to be confined to the Gulf Coast states from Louisiana to Florida, and specimens from Mineral County reported as *P. shubuta* were probably *Perlesta ephelida* (Grubbs and DeWalt 2012). *Sweltsa onkos* was separated into two species by Kondratieff and Kirchner (2009), so that most specimens previously described as *S. onkos* in West Virginia would now be considered *S. hoffmani*. The presence of *Isoperla gibbsae* in West Virginia is supported by a single literature record (Kirchner 1978), and we have not recovered this species in museums or in the field. The taxonomy of the large genus *Isoperla* was restructured by Szczytko and Kondratieff (2015) and continues to be in a state of flux (e.g. Verdone and Kondratieff (2017), Grubbs et al. (2025)). Thus, many earlier *Isoperla* identifications have been rendered suspect, with older specimens often being more accurately assigned to a different species, such as *Isoperla kirchneri* Szczytko & Kondratieff, 2015 or *Isoperla pseudosimilis* Szczytko & Kondratieff, 2015. It should be noted that large stream taxa, including many perlids and some perlodids, are relatively under-represented in this dataset. Insufficient attention has so far been given to collecting adults from these habitats.

### Data methods

The Illinois Natural History Survey Insect Collection (INHS-IC) uses the collection management software TaxonWorks to manage its specimen records (TaxonWorks Community 2022). The INHS-IC began with a total of 750 unique stonefly records from West Virginia through August 2023. Over the following two years, 3,214 records were added to the database through the digitization of fresh material and specimens borrowed from Brigham Young University, Colorado State University, the United States National Museum and West Virginia University. Additional specimen records were shared by other institutions and individual researchers (Table 1). Although TaxonWorks divides sexes and life stages with the same catalog number into separate rows, we use the term “record” to refer to the contents of one vial or to one pinned specimen. One literature record includes all specimens of the same species collected during one collecting event. Institution codes were copied from the Global Registry of Scientific Collections when possible (GBIF 2025).

The data were cleaned using Microsoft Excel and OpenRefine (Delpeuch et al. 2025) to eliminate duplicates and reduce misspellings. Most specimens were provided with catalog numbers from their parent institution. Records without unique identifiers were assigned new UUIDs for ease of searchability. Five custom fields were added to the dataset using publicly available shapefiles and TIFF files from the USEPA and USGS: Elevation Join (m), HUC6, HUC8, Ecoregion Level III and Ecoregion Level IV (USGS 2023, WVGIS 2025, USEPA 2025). Data from these layers were joined to the dataset using the Spatial Join and Pairwise Intersect tools in ArcGIS Pro (Esri 2023).

## Geographic coverage

### Description

**Figure 1.**
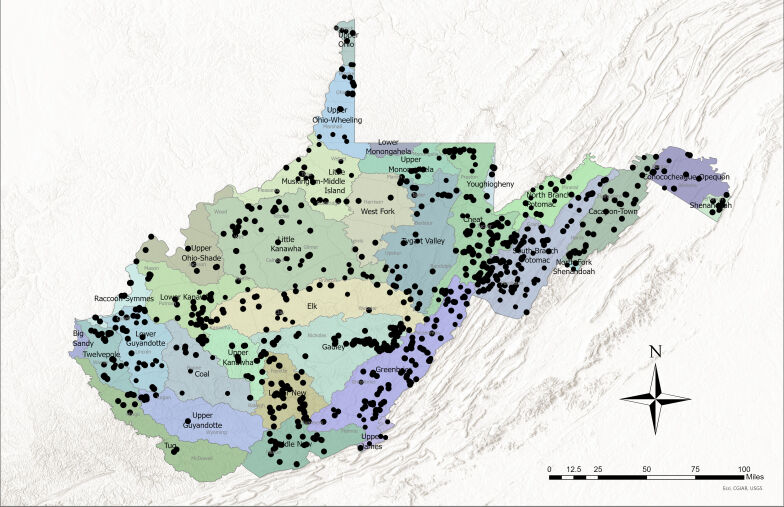
A map of all unique sampling locations, overlaid on 32 HUC8 watersheds.

The United States Environmental Protection Agency divides West Virginia into four Level III Ecoregions and fifteen Level IV Ecoregions, to account for differences in elevation, landcover and climatic variables. The United States Geological Survey has designated nine six-digit hydrologic units (HUC6s) that overlap with state boundaries. These are further subdivided into 32 HUC8 hydrologic units (Fig. 1). Our dataset includes separate columns for these four categories to facilitate future geospatial analyses. The only two West Virginian HUC8s omitted from the dataset are the Big Sandy and Lower Monongahela watersheds, both of which lack specimen records at this time.

### Coordinates

37.2°N and 40.6°N Latitude; 82.6°W and 77.7°W Longitude.

## Taxonomic coverage

### Description

Nine Plecopteran families are present in West Virginia. We have recorded 150 species, 11 of which are reported for the first time (Table 2). The West Virginia Department of Natural Resources has listed 11 of these species as Species of Greatest Conservation Need (SGCN) at the state level (WVDNR 2015). The Northeast Association of Fish and Wildlife Agencies has designated 16 of these species as Regional Species of Greatest Conservation Need (RSGCN), including two of the new state records (Northeast Fish and Wildlife Diversity Technical Committee 2024).

## Temporal coverage

**Data range:** 1891-9-16 – 2026-1-22.

### Notes

**Figure 2.**
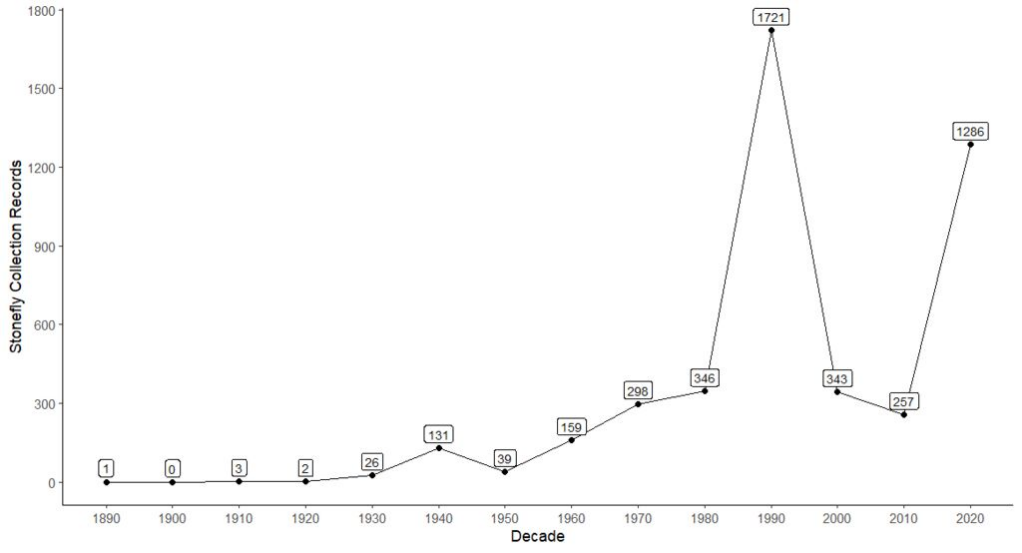
Stonefly collection in West Virginia by decade according to our dataset.

This dataset documents more than a century of stonefly collection in West Virginia. The earliest record was collected at Harpers Ferry in 1891 by an unknown collector. More systematic collecting began in 1930 and continued steadily until the 1990s (Fig. 2). Researchers from Marshall University collected hundreds of stonefly records during the 1960s and 1970s (Tarter and United States Army Corps of Engineers 1976). Since most of their specimens have not survived, we did not include their specimen records here. The peak of collection activity occurred in the 1990s, after which efforts declined until 2017, when the authors from the Illinois Natural History Survey and Western Kentucky University commenced sampling for species of conservation concern.

## Usage licence

### Usage licence

Open Data Commons Attribution License

## Data resources

### Data package title

Plecoptera of West Virginia

### Resource link

https://doi.org/10.15468/yhydx6

### Number of data sets

1

### Data set 1.

#### Data set name

Plecoptera of West Virginia

#### Data format

DarwinCore Archive

#### Character set

UTF-8

#### Description

This dataset includes 4,612 specimen records divided into 6,267 rows (Cook et al. 2026, Suppl. material 1). It brings together historical specimens from museums and other collections, recently collected records by the authors and records by trusted literature sources. All records have been georeferenced, and all but 204 have been provided with catalog numbers. The dataset is divided into 72 columns. A description of each column can be found in Table 3. The dataset is divided into 72 columns, named and structured in accordance with standards recommended by the Darwin Core Maintenance Group (Darwin Core Maintenance Group 2021).

## Supplementary Material

6B789B85-59ED-594C-A17A-107F3CC16D89 10.3897/BDJ.14.e185229.suppl1 Supplementary material 1 Plecoptera of West Virginia: Version 1.1 Data type occurrences Brief description This dataset contains records of 4,612 individual collection objects divided into 6,267 rows of specimen data from the state of West Virginia. Specimens have been gathered from 55 counties, 30 HUC8 drainages and 14 Level IV ecoregions, over a period of 134 years. All specimens have been identified either to genus or species level. All records have been given geographic coordinates by their collector or by a curator and all but 204 have been provided with catalog numbers. The dataset is divided into 72 columns, named and structured in accordance with standards recommended by the Darwin Core Maintenance Group. This dataset was last updated in February 2026. File: oo_1585048.csv https://binary.pensoft.net/file/1585048 Cook TZ, DeWalt RE, Grubbs SA, Hogan PN, Hart LV, Burton DK, Clark SM, Baumann RW, Myers LW

## Figures and Tables

**Table 1.** Dataset records by institution.

**Institution Code**	**Institution Name**	**# of records**	**# of specimens**	**Temporal range**
INHS	Illinois Natural History Survey Insect Collection, Champaign, IL, USA	1743	6733	1936-2026
BYUC	Monte L. Bean Life Science Museum, Provo, UT, USA	1361	11547	1924-2016
CSUIC	C. P. Gillette Museum of Arthropod Diversity, Ft. Collins, CO, USA	365	2281	1971-2017
USNMENT	United States National Museum, Entomology Collections, Washington, D.C., USA	321	3461	1891-2016
WVDEP	West Virginia Department of Environmental Protection, Charleston, WV, USA	260	1011	1990-2024
WVUC	West Virginia University, Morgantown, WV, USA	165	1155	1989-1991
WKUC	Western Kentucky University, Bowling Green, KY, USA	93	504	1982-2018
UKIC	University of Kentucky Insect Collection, Lexington, KY, USA	69	509	1958-1980
CNCI	Canadian National Collection, Ottawa, ON, Canada	47	298	1940-1991
CM-IZ	Carnegie Museum of Natural History, Pittsburgh, PA, USA	13	13	1910-1994
CUIC	Cornell University Insect Collection, Ithaca, NY	6	45	1930
OSUC	The Ohio State University Museum of Biological Diversity, Columbus, OH	1	6	1966
PNHC	Phillip N. Hogan Personal Collection	61	346	2021-2022
BPSC	Bill P. Stark Personal Collection	16	62	1978-1999
CHNC	Charles H. Nelson Personal Collection	14	176	1976-2006
RFKC	R. F. Kirchner Personal Collection	13	40	1973-1995
DCTC	Donald C. Tarter Personal Collection	5	12	2008-2011
Literature records		26	192	1973-2005

**Table 2.** A checklist of stoneflies of West Virginia, based on our dataset. The symbol "*" signifies a new West Virginia state record. The third column indicates whether a species has been marked as a Species of Greatest Conservation Need (SGCN). The initials "SGCN" indicate that a species has been listed by West Virginia as an SGCN. "RSGCN" indicates that the species has been listed by the Northeast Association of Fish and Wildlife Agencies as a Region (-scale) SGCN.

Family	Scientific Name	SGCN status	Unique localities
Capniidae	*Allocapnia aurora* Ricker, 1952*	None	1
Capniidae	*Allocapnia curiosa* Frison, 1942	None	26
Capniidae	*Allocapnia forbesi* Frison, 1929	None	5
Capniidae	*Allocapnia frisoni* Ross & Ricker, 1964	None	30
Capniidae	*Allocapnia frumi* Kirchner, 1982	SGCN+RSGCN	16
Capniidae	*Allocapnia granulata* (Claassen, 1924)	None	57
Capniidae	*Allocapnia harperi* Kirchner, 1980	RSGCN	17
Capniidae	*Allocapnia illinoensis* Frison, 1935	RSGCN	1
Capniidae	*Allocapnia loshada* Ricker, 1952	None	2
Capniidae	*Allocapnia maria* Hanson, 1942	None	11
Capniidae	*Allocapnia mystica* Frison, 1929	None	9
Capniidae	*Allocapnia nivicola* (Fitch, 1847)	None	91
Capniidae	*Allocapnia ohioensis* Ross & Ricker, 1964	None	5
Capniidae	*Allocapnia pygmaea* (Burmeister, 1839)	None	89
Capniidae	*Allocapnia recta* (Claassen, 1924)	None	110
Capniidae	*Allocapnia rickeri* Frison, 1942	None	114
Capniidae	*Allocapnia simmonsi* Kondratieff & Voshell, 1981	RSGCN	5
Capniidae	*Allocapnia vivipara* (Claassen, 1924)	None	24
Capniidae	*Allocapnia zola* Ricker, 1952	None	24
Capniidae	*Paracapnia angulata* Hanson, 1961	None	67
Capniidae	*Paracapnia opis* (Newman, 1839)*	None	2
Chloroperlidae	*Alloperla aracoma* Harper & Kirchner, 1978	SGCN+RSGCN	3
Chloroperlidae	*Alloperla biserrata* Nelson & Kondratieff, 1980	SGCN+RSGCN	2
Chloroperlidae	*Alloperla chloris* Frison, 1934	None	8
Chloroperlidae	*Alloperla clarki* Grubbs & Baumann, 2019	None	2
Chloroperlidae	*Alloperla concolor* Ricker, 1935	None	4
Chloroperlidae	*Alloperla idei* (Ricker, 1935)	None	1
Chloroperlidae	*Alloperla imbecilla* (Say, 1823)	None	24
Chloroperlidae	*Alloperla petasata* Surdick, 2004	None	7
Chloroperlidae	*Alloperla usa* Ricker, 1952	None	23
Chloroperlidae	*Haploperla brevis* (Banks, 1895)	None	69
Chloroperlidae	*Rasvena terna* (Frison, 1942)	None	7
Chloroperlidae	*Suwallia marginata* (Banks, 1897)	None	13
Chloroperlidae	*Sweltsa hoffmani* Kondratieff & Kirchner, 2009	None	62
Chloroperlidae	*Sweltsa lateralis* (Banks, 1911)	None	36
Chloroperlidae	*Sweltsa naica* (Provancher, 1876)	None	6
Chloroperlidae	*Sweltsa palearata* Surdick, 2004	RSGCN	5
Chloroperlidae	*Sweltsa pocahontas* Kirchner & Kondratieff, 1988	SGCN+RSGCN	13
Chloroperlidae	*Utaperla gaspesiana* Harper & Roy, 1975	SGCN+RSGCN	1
Leuctridae	*Leuctra alexanderi* Hanson, 1941	None	58
Leuctridae	*Leuctra biloba* Claassen, 1923	None	2
Leuctridae	*Leuctra carolinensis* Claassen, 1923	None	17
Leuctridae	*Leuctra duplicata* Claassen, 1923	None	48
Leuctridae	*Leuctra ferruginea* (Walker, 1852)	None	39
Leuctridae	*Leuctra grandis* Banks, 1906	None	39
Leuctridae	*Leuctra maria* Hanson, 1941	None	3
Leuctridae	*Leuctra rickeri* James, 1976	None	2
Leuctridae	*Leuctra sibleyi* Claassen, 1923	None	48
Leuctridae	*Leuctra tenella* Provancher, 1878	None	17
Leuctridae	*Leuctra tenuis* (Pictet, 1841)	None	16
Leuctridae	*Leuctra triloba* Claassen, 1923	None	1
Leuctridae	*Leuctra truncata* Claassen, 1923	None	3
Leuctridae	*Leuctra variabilis* Hanson, 1941*	None	4
Leuctridae	*Megaleuctra flinti* Baumann, 1973	SGCN+RSGCN	14
Leuctridae	*Paraleuctra sara* (Claassen, 1937)	None	69
Leuctridae	*Zealeuctra claasseni* (Frison, 1929)	None	1
Leuctridae	*Zealeuctra fraxina* Ricker & Ross, 1969	None	1
Nemouridae	*Amphinemura delosa* (Ricker, 1952)	None	26
Nemouridae	*Amphinemura nigritta* (Provancher, 1876)	None	103
Nemouridae	*Amphinemura wui* (Claassen, 1936)	None	38
Nemouridae	*Nemoura arctica* Esben-Petersen, 1910	None	2
Nemouridae	*Ostrocerca albidipennis* (Walker, 1852)	None	40
Nemouridae	*Ostrocerca complexa* (Claassen, 1937)	SGCN	28
Nemouridae	*Ostrocerca prolongata* (Claassen, 1923)	SGCN+RSGCN	6
Nemouridae	*Ostrocerca truncata* (Claassen, 1923)	None	37
Nemouridae	*Paranemoura perfecta* (Walker, 1852)	None	30
Nemouridae	*Prostoia completa* (Walker, 1852)	None	13
Nemouridae	*Prostoia similis* (Hagen, 1861)	None	39
Nemouridae	*Soyedina carolinensis* (Claassen, 1923)	None	4
Nemouridae	*Soyedina merritti* Baumann & Grubbs, 1996*	RSGCN	2
Nemouridae	*Soyedina vallicularia* (Wu, 1923)	None	12
Nemouridae	*Soyedina washingtoni* (Claassen, 1923)	None	11
Perlidae	*Acroneuria abnormis* (Newman, 1838)	None	72
Perlidae	*Acroneuria arenosa* (Pictet, 1841)	None	3
Perlidae	*Acroneuria carolinensis* (Banks, 1905)	None	87
Perlidae	*Acroneuria filicis* Frison, 1942	None	4
Perlidae	*Acroneuria frisoni* Stark & Brown, 1991	None	11
Perlidae	*Acroneuria internata* (Walker, 1852)	None	4
Perlidae	*Acroneuria kosztarabi* Kondratieff & Kirchner, 1993	None	4
Perlidae	*Acroneuria lycorias* (Newman, 1839)	None	9
Perlidae	*Agnetina annulipes* (Hagen, 1861)	None	9
Perlidae	*Agnetina capitata* (Pictet, 1841)	None	5
Perlidae	*Agnetina flavescens* (Walsh, 1862)	None	6
Perlidae	*Eccoptura xanthenes* (Newman, 1838)	None	31
Perlidae	*Hansonoperla appalachia* Nelson, 1979	SGCN+RSGCN	19
Perlidae	*Hansonoperla hokolesqua* Kondratieff & Kirchner, 1996	SGCN+RSGCN	1
Perlidae	*Neoperla carlsoni* Stark & Baumann, 1978*	None	3
Perlidae	*Neoperla catharae* Stark & Baumann, 1978*	None	5
Perlidae	*Neoperla choctaw* Stark & Baumann, 1978	None	1
Perlidae	*Neoperla clymene* (Newman, 1839)	None	2
Perlidae	*Neoperla coosa* Smith & Stark, 1998*	None	7
Perlidae	*Neoperla occipitalis* (Pictet, 1841)*	None	2
Perlidae	*Neoperla robisoni* Poulton & Stewart, 1986	None	2
Perlidae	*Neoperla stewarti* Stark & Baumann, 1978	None	7
Perlidae	*Paragnetina immarginata* (Say, 1823)	None	12
Perlidae	*Paragnetina media* (Walker, 1852)	None	9
Perlidae	*Perlesta decipiens* (Walsh, 1862)	None	2
Perlidae	*Perlesta ephelida* Grubbs & DeWalt, 2012	None	5
Perlidae	*Perlesta nelsoni* Stark, 1989	None	2
Perlidae	*Perlesta placida* (Hagen, 1861)	None	11
Perlidae	*Perlesta teaysia* Kirchner & Kondratieff, 1997	None	8
Perlidae	*Perlinella drymo* (Newman, 1839)	None	1
Perlidae	*Perlinella ephyre* (Newman, 1839)	None	5
Perlodidae	*Clioperla clio* (Newman, 1839)	None	29
Perlodidae	*Cultus verticalis* (Banks, 1920)	None	4
Perlodidae	*Diploperla duplicata* (Banks, 1920)	None	5
Perlodidae	*Diploperla kanawholensis* Kirchner & Kondratieff, 1984	SGCN+RSGCN	10
Perlodidae	*Diploperla robusta* Stark & Gaufin, 1974	None	29
Perlodidae	*Helopicus subvarians* (Banks, 1920)	None	5
Perlodidae	*Isogenoides hansoni* (Ricker, 1952)	None	11
Perlodidae	*Isoperla bilineata* (Say, 1823)	None	1
Perlodidae	*Isoperla burksi* Frison, 1942	None	2
Perlodidae	*Isoperla dicala* Frison, 1942	None	14
Perlodidae	*Isoperla evanescens* Verdone & Kondratieff, 2016	None	3
Perlodidae	*Isoperla holochlora* Klápalek, 1923	None	6
Perlodidae	*Isoperla kirchneri* Szczytko & Kondratieff, 2015	None	21
Perlodidae	*Isoperla lata* Frison, 1942	None	4
Perlodidae	*Isoperla montana* (Banks, 1898)	None	8
Perlodidae	*Isoperla myersi* Szczytko & Kondratieff, 2015*	RSGCN	5
Perlodidae	*Isoperla nana* (Walsh, 1862)*	None	1
Perlodidae	*Isoperla nelsoni* Szczytko & Kondratieff, 2015	None	13
Perlodidae	*Isoperla orata* Frison, 1942	None	1
Perlodidae	*Isoperla pseudolata* Szczytko & Kondratieff, 2015	None	3
Perlodidae	*Isoperla pseudosimilis* Szczytko & Kondratieff, 2015	None	35
Perlodidae	*Isoperla richardsoni* Frison, 1935	None	1
Perlodidae	*Malirekus hastatus* (Banks, 1920)	None	28
Perlodidae	*Malirekus iroquois* Stark & Szczytko, 1988*	None	3
Perlodidae	*Remenus bilobatus* (Needham & Claassen, 1925)	None	13
Perlodidae	*Yugus bulbosus* (Frison, 1942)	None	4
Perlodidae	*Yugus kirchneri* Nelson, 2001	None	40
Peltoperlidae	*Peltoperla arcuata* Needham, 1925	None	24
Peltoperlidae	*Peltoperla tarteri* Stark & Kondratieff, 1987	None	6
Peltoperlidae	*Tallaperla maria* (Needham & Smith, 1916)	None	39
Pteronarcyidae	*Pteronarcys biloba* Newman, 1838	None	10
Pteronarcyidae	*Pteronarcys comstocki* Smith, 1917	None	6
Pteronarcyidae	*Pteronarcys dorsata* (Say, 1923)	None	7
Pteronarcyidae	*Pteronarcys proteus* Newman, 1838	None	55
Taeniopterigidae	*Bolotoperla rossi* (Frison, 1942)	None	14
Taeniopterigidae	*Oemopteryx bimaculata* Verdone, Williams, Beaty & Holland, 2025	None	2
Taeniopterigidae	*Oemopteryx contorta* (Needham & Claassen, 1925)	None	13
Taeniopterigidae	*Oemopteryx glacialis* (Barnston, 1848)	None	9
Taeniopterigidae	*Strophopteryx appalachia* Ricker & Ross, 1975	None	20
Taeniopterigidae	*Strophopteryx fasciata* (Burmeister, 1839)	None	70
Taeniopterigidae	*Taenionema atlanticum* Ricker & Ross, 1975	None	35
Taeniopterigidae	*Taeniopteryx burksi* Ricker & Ross 1968	None	80
Taeniopterigidae	*Taeniopteryx lita* Frison, 1942	None	3
Taeniopterigidae	*Taeniopteryx maura* (Pictet, 1841)	None	57
Taeniopterigidae	*Taeniopteryx metequi* Ricker & Ross, 1968	None	22
Taeniopterigidae	*Taeniopteryx parvula* Banks, 1918	None	19
Taeniopterigidae	*Taeniopteryx ugola* Ricker & Ross, 1968	None	25

**New State Records**

**
Capniidae
**

***Allocapnia aurora* Ricker, 1952** *-* **Doddridge Co**.: Wilhelm Run at Central Station, [39.2935, -80.8275], 21 February 1996, S. M. Clark, 34♂ (BYUC 167847).

***Paracapnia opis* (Newman, 1839)** - **Randolph Co**.: Shavers Fork of Cheat River, Bowden, [38.9083, -79.71345], 4-IV-1995, S. M. Clark, 1♂ (BYUC 168449). Shavers Fork, along WV-250, Monongahela NF, 38.62493, -79.86842, 1085 m a.s.l., 2025-III-24, T. Z. Cook, P. N. Hogan, TZC-2025-027, beating sheet, 33♂ (INHS Insect Collection 1544759, 1544760, 1544761, 1544762, 1544763).

**
Leuctridae
**

***Leuctra variabilis* Hanson, 1941** - **Pendleton Co**., Gulf Run, at Whites Run Rd., Monongahela NF, 38.85801, -79.47812, 671 m a.s.l., 14-X-2023, R. E. DeWalt, P. N. Hogan, T. Z. Cook, RED-2023-103B, beating sheet, 4♂ (INHS Insect Collection 1523443, INHS Insect Collection 1523449, PNHC_0001399, PNHC_0001457)**. Randolph Co**., Spruce Knob Lake, [38.70266, -79.58816], 28-IX-2002, L. T. Miller, 7♂, 8♀ (BYUC 168767).

**
Nemouridae
**

***Soyedina merritti* Baumann & Grubbs, 1996** - **Preston Co**., trib. Lick Creek, Hemlock Tr., Coopers Rock SF, 39.65509, -79.73604, 506 m a.s.l., 11-III-2024, T. Z. Cook, R. E. DeWalt, TZC-2024-007, beating sh. 3♂, 1♀(INHS Insect Collection 1524125, 1524126, 1524127, 1524128). Tributary Glades Run, University For., 39.67111, -79.77631, 640 m a.s.l., 12-III-2024, T. Z. Cook, R. E. DeWalt, TZC-2024-011, beating sh., 15♂, 8♀ (INHS Insect Collection 1524140, 1524141, 1524142, 1524143, 1524144, 1524145, 1524146).

**
Perlidae
**

***Neoperla carlsoni* Stark & Baumann, 1978** - **Jefferson Co**., Shenandoah River near Shannondale, [39.230705, -77.80973], 24-VII-1993, S. M. Clark, 5♀ (BYUC 169016). **Pendleton Co**., Smoke Hole Camp, [38.89571, -79.28042], 28-29 Aug 1963, R. & O. Flint, 1♂ (USNMENT 01092028). **Summers Co**. Indian Creek, Rt 33/2, [37.5213, -80.81469], 14 Sept 1982, B. C. Kondratieff, 1♀ (CSU_ENT 5000444).

***Neoperla catharae* Stark & Baumann, 1978** - **Fayette Co**., New River, Stonecliff, [37.933384, -81.06306], 1-VIII-1992, S. M. Clark, 1♂, 1♀ (BYUC 169049). **Hampshire Co**., South Branch Potomac River, Grace, [39.44141, -78.67097], 21-VIII-1997, S. M. Clark, 2♂, 3♀ (BYUC 168036). **Jefferson Co**., Shenandoah River near Shannondale, [39.230705, -77.80973], 24-VII-1993, S. M. Clark, 3♂ (BYUC 169015). **Pendleton Co**., Smoke Hole, [38.8578, -79.2813], 7 Aug. 1930, P. W. Claassen, 14♂, 11♀ (CUIC 000071383, 000071384, 000071386). Smoke Hole Camp[ground], [38.89571, -79.28042], 28-29 Aug., 1963, R. & O. Flint, 1♂ (USNMENT 01092029). **Summers Co**., New River at Sandstone, [37.77351, -80.89101], 27-VIII-1991, S. M. Clark, 1♂, 2♀ (BYUC 168035).

***Neoperla coosa* Smith & Stark, 1998** - **Fayette Co**., New River, Stonecliff, [37.933384, -81.06306], 1-VIII-1992, S. M. Clark 4♂, 4♀ (BYUC 168024). New River at Fayette Station, [38.066432, -81.08180], 14-VII-1994, S. M. Clark, 2♂, 4♀ (BYUC 168034). New River at Fayette Station, 26 June 1996, S. M. Clark, 1♂, 4♀ (BYUC 167671). **Raleigh Co**., New River, nr. Grandview St Park, [37.83289, -81.05498], 14 July 1982, B. C. Kondratieff 1♀ (CSU_ENT 5000443). New River at Terry Junction, [37.85053, -81.08185], 10-VI-1994, S. M. Clark, 6♂ (BYUC 167674). New River at Grandview Sand Bar, near Terry Junction, [37.83289, -81.05498], 13 Aug. 1998, S. M. Clark, 2♀ (BYUC 167940). **Summers Co**., Indian Creek, Indian Creek Campgrd, [37.52823, -80.82703], 6 July 1982, B. C. Kondratieff, 1♂, 3♀ (CSU_ENT 5000453). Bluestone River, at Gorge, Pipestem State Park, [37.53844, -81.00792], 14 July 1982, B. C. Kondratieff, 6♂, 2♀, 1N (CSU_ENT 5000445, 5000446). New River, at Hinton, [37.67517, -80.89253], at light, 26 July 1982, B. C. Kondratieff (CSU_ENT 5000449). New River at Sandstone, [37.77371, -80.89257], 10-VII-2001, S. M. Clark, 1♂, 2♀ (BYUC 168956).

***Neoperla occipitalis* (Pictet, 1841)** – **Mercer Co**., mouth of Brush Creek, [37.47762, -81.06144], July 18, 1930, P. W. Claassen, 1♀ (CUIC 000071394). **Tucker Co**., Blackwater River, 19-VII-2022, P. N. Hogan, PNH-2022-132, UV light, 1♂, (INHS Insect Collection 1523747).

**
Perlodidae
**

***Isoperla myersi* Szczytko & Kondratieff, 2015** - **Fayette Co**., Cathedral Falls, Laurel Branch, Hwy 60, jct. New River 1 mi E Gauley Bridge, [38.15397, -81.18002], 21-V-1990, Baumann & Clark, 1♀ (BYUC 169068). **Preston Co**., Terra Alta, 1049 Feather Rd., Malaise Trap, 796 m a.s.l., 21 July-3 Aug 2016, N 39.5411° W 79.4951°, S. Scheffer, M. Lewis, 1♀ (USNMENT 02032565). **Mingo Co**., Laurel Fork of Pigeon Creek, [37.85439, -82.18148], 25-V-1975, Coll: R. F. Kirchner, em: 25-V-75, 2♂, 2♀, 6N, 4 exv. (CSU_ENT 5000559). **Raleigh Co**., Beaver Creek, Rt 19/13, [37.72728, -81.13189] 23 June 1983, B. C. Kondratieff, 2♀ (CSU_ENT 5000452). **Randolph Co**., Elk R., 3.2 km E Samp along CR-49, 38.53772, -80.15522, 695 m a.s.l., 13 June 2017, R. E. DeWalt, E. D. Snyder, J. B. Beinlich, RED-2017-044, beating sh., 1♂, 3♀ (INHS Insect Collection 1544664, 1544665, 1544666, 1544667).

***Isoperla nana* (Walsh, 1862)** - **Mineral Co**., New Creek at New Creek, [39.37704, -79.02581], 26-V-1993, S. M. Clark, 1♂ (BYUC 169045).

***Malirekus iroquois* Stark & Szczytko, 1988** - **Grant Co**., 11 km SE Petersburg, Elkhorn Mountain, [38.91615, -79.07887], 22-IV-1992, R. Acciavatti 1♂, (CM-IZ) [no catalog number]. **Pocahontas Co**., Marlinton, [38.22429, -80.09606], 14 May 1990, B. C. Kondratieff, J. L. Welch, R. F. Kirchner, 1♀ (CSU_ENT 3001627). Hills Cr. Scenic Area, off Rt 39, [38.17557, -80.33617], 14 May 1990, B. C. Kondratieff, J. L. Welch, R. F. Kirchner, 1♀ (CSU_ENT 3001628).

**Table 3.** Dataset metadata.

Column Heading	Description
basisOfRecord	The specific nature of the data record.
occurrenceID	A UUID for the dwc:Occurrence (as opposed to a particular digital record of the dwc:Occurrence).
occurrenceIDSource	Custom Field - The institution or person who assigned the UUID.
catalogNumber	An identifier (preferably unique) for the record within the dataset or collection.
individualCount	The number of individuals present at the time of the dwc:Occurrence.
preparations	A list (concatenated and separated) of preparations and preservation methods for a dwc:MaterialEntity.
lifeStage	The age class or life stage of the dwc:Organism(s) at the time the dwc:Occurrence was recorded.
sex	The sex of the biological individual(s) represented in the dwc:Occurrence.
continent	The name of the continent in which the dcterms:Location occurs.
country	The name of the country or major administrative unit in which the dcterms:Location occurs.
stateProvince	The name of the next smaller administrative region than country (state, province, canton, department, region, etc.) in which the dcterms:Location occurs.
county	The full, unabbreviated name of the next smaller administrative region than stateProvince (county, shire, department, etc.) in which the dcterms:Location occurs.
eventDate	The date-time or interval during which a dwc:Event occurred. For occurrences, this is the date-time when the dwc:Event was recorded.
year	The four-digit year in which the dwc:Event occurred.
month	The integer month in which the dwc:Event occurred.
day	The integer day of the month on which the dwc:Event occurred.
startDayOfYear	The earliest integer day of the year on which the dwc:Event occurred (1 for January 1, 365 for December 31, except in a leap year, in which case it is 366).
endDayOfYear	The latest integer day of the year on which the dwc:Event occurred (1 for January 1, 365 for December 31, except in a leap year, in which case it is 366).
fieldNumber	An identifier given to the dwc:Event in the field. Often serves as a link between field notes and the dwc:Event.
samplingProtocol	The names of, references to, or descriptions of the methods or protocols used during a dwc:Event.
habitat	A category or description of the habitat in which the dwc:Event occurred.
verbatimElevation	The original description of the elevation (altitude, usually above sea level) of the Location.
Elevation Join (m)	Custom field - the elevation in meters above sea level of the coordinate pair according to a 1-meter statewide Digital Elevation Model.
verbatimEventDate	The verbatim original representation of the date and time information for a dwc:Event.
verbatimLocality	The original textual description of the place.
waterBody	The name of the water body in which the dcterms:Location occurs.
recordedBy	A list of people responsible for recording the original dwc:Occurrence.
recordedByID	A list (concatenated and separated) of the globally unique identifier for the person, people, groups, or organizations responsible for recording the original dwc:Occurrence. ORCID identifiers are used when possible.
identifiedBy	A list (concatenated and separated) of names of people, groups, or organizations who assigned the dwc:Taxon to the subject.
identifiedByID	A list (concatenated and separated) of the globally unique identifier for the person, people, groups, or organizations responsible for assigning the dwc:Taxon to the subject.
dateIdentified	The date on which the subject was determined as representing the dwc:Taxon.
nomenclaturalCode	The nomenclatural code (or codes in the case of an ambiregnal name) under which the dwc:scientificName is constructed.
kingdom	The full scientific name of the kingdom in which the dwc:Taxon is classified.
phylum	The full scientific name of the phylum or division in which the dwc:Taxon is classified.
class	The full scientific name of the class in which the dwc:Taxon is classified.
order	The full scientific name of the order in which the dwc:Taxon is classified.
higherClassification	A list (concatenated and separated) of taxa names terminating at the rank immediately superior to the referenced dwc:Taxon.
family	The full scientific name of the family in which the dwc:Taxon is classified.
genus	The full scientific name of the genus in which the dwc:Taxon is classified.
specificEpithet	The name of the first or species epithet of the dwc:scientificName.
scientificName	The full scientific name, with authorship and date information if known. Names have been taken to the lowest taxonomic rank possible and all names in this dataset are in keeping with the International Commission on Zoological Nomenclature.
scientificNameAuthorship	The authorship information for the dwc:scientificName formatted according to the conventions of the applicable dwc:nomenclaturalCode.
taxonRank	The taxonomic rank of the most specific name in the dwc:scientificName.
typeStatus	A list (concatenated and separated) of nomenclatural types (type status, typified scientific name, publication) applied to the subject.
institutionCode	The name (or acronym) in use by the institution having custody of the object(s) or information referred to in the record.
InstitutionID	An identifier for the institution having custody of the object(s) or information referred to in the record.
verbatimCoordinates	The verbatim original spatial coordinates of the dcterms:Location.
verbatimLatitude	The verbatim original latitude of the dcterms:Location.
verbatimLongitude	The verbatim original longitude of the dcterms:Location.
verbatimSRS	The ellipsoid, geodetic datum, or spatial reference system (SRS) upon which coordinates given in dwc:verbatimLatitude and dwc:verbatimLongitude, or dwc:verbatimCoordinates are based.
decimalLatitude	The geographic latitude (in decimal degrees, using the spatial reference system given in dwc:geodeticDatum) of the geographic center of a dcterms:Location. Positive values are north of the Equator, negative values are south of it. Legal values lie between -90 and 90, inclusive.
decimalLongitude	The geographic longitude (in decimal degrees, using the spatial reference system given in dwc:geodeticDatum) of the geographic center of a dcterms:Location. Positive values are east of the Greenwich Meridian, negative values are west of it. Legal values lie between -180 and 180, inclusive.
geodeticDatum	The ellipsoid, geodetic datum, or spatial reference system (SRS) upon which the geographic coordinates given in dwc:decimalLatitude and dwc:decimalLongitude are based.
footprintWKT	A Well-Known Text (WKT) representation of the shape (footprint, geometry) that defines the dcterms:Location. A dcterms:Location may have both a point-radius representation (see dwc:decimalLatitude) and a footprint representation, and they may differ from each other.
coordinateUncertaintyInMeters	The horizontal distance (in meters) from the given dwc:decimalLatitude and dwc:decimalLongitude describing the smallest circle containing the whole of the dcterms:Location.
georeferenceProtocol	A description or reference to the methods used to determine the spatial footprint, coordinates, and uncertainties.
georeferenceRemarks	Comments or notes about the spatial description determination, explaining assumptions made in addition or opposition to the those formalized in the method referred to in dwc:georeferenceProtocol.
georeferenceSources	A description of the source of the georeference.
georeferencedBy	The name of person who determined the georeference (spatial representation) for the dcterms:Location.
georeferencedDate	The date on which the dcterms:Location was georeferenced.
occurrenceStatus	A statement about the presence or absence of a dwc:Taxon at a dcterms:Location.
associatedMedia	A list (concatenated and separated) of identifiers (publication, global unique identifier, URI) of media associated with the dwc:Occurrence.
occurrenceRemarks	Comments or notes about the dwc:Occurrence.
eventRemarks	Comments or notes about the dwc:Event.
verbatimLabel	The verbatim text from the specimen labels. Labels are concatenated and separated.
TW:DataAttribute:CollectionObject:Emerged Date	Custom attribute field associated with some records in TaxonWorks (database): the observed date of emergence of a reared specimen(s).
TW:DataAttribute:CollectionObject:Park	Custom attribute field associated with some records in TaxonWorks (database): the name of the public forest, park, or wildlife management area in which the specimen(s) were collected.
associatedReferences	A list (concatenated and separated) of identifiers (publication, bibliographic reference, global unique identifier, URI) of literature associated with the dwc:Occurrence.
HUC6	Custom Field - the Level 6 Hydrologic Unit code in which the coordinate pair is located.
HUC8	Custom Field - the Level 8 Hydrologic Unit code in which the coordinate pair is located.
Ecoregion Level III	Custom Field - the Level III EPA ecoregion in which the coordinate pair is located.
Ecoregion Level IV	Custom Field - the Level IV EPA ecoregion in which the coordinate pair is located.
